# Cellular Metabolomics Profiles Associated With Drug Chemosensitivity in AML

**DOI:** 10.3389/fonc.2021.678008

**Published:** 2021-06-10

**Authors:** Bradley Stockard, Neha Bhise, Miyoung Shin, Joy Guingab-Cagmat, Timothy J. Garrett, Stanley Pounds, Jatinder K. Lamba

**Affiliations:** ^1^ Department of Pharmacotherapy and Translational Research, College of Pharmacy, University of Florida, Gainesville, FL, United States; ^2^ Southeast Center for Integrated Metabolomics, University of Florida, Gainesville, FL, United States; ^3^ Department of Biostatistics, St Jude Children’s Research Hospital, Memphis, TN, United States; ^4^ University of Florida Health Cancer Center, Gainesville, FL, United States; ^5^ Center for Pharmacogenetics, University of Florida, Gainesville, FL, United States

**Keywords:** leukemia, AML, drug resistance, cytarabine, doxorubicin, metabolomics

## Abstract

**Background:**

Acute myeloid leukemia (AML) is a hematological malignancy with a dismal prognosis. For over four decades, AML has primarily been treated by cytarabine combined with an anthracycline. Although a significant proportion of patients achieve remission with this regimen, roughly 40% of children and 70% of adults relapse. Over 90% of patients with resistant or relapsed AML die within 3 years. Thus, relapsed and resistant disease following treatment with standard therapy are the most common clinical failures that occur in treating this disease. In this study, we evaluated the relationship between AML cell line global metabolomes and variation in chemosensitivity.

**Methods:**

We performed global metabolomics on seven AML cell lines with varying chemosensitivity to cytarabine and the anthracycline doxorubicin (MV4.11, KG-1, HL-60, Kasumi-1, AML-193, ME1, THP-1) using ultra-high performance liquid chromatography – mass spectrometry (UHPLC-MS). Univariate and multivariate analyses were performed on the metabolite peak intensity values from UHPLC-MS using MetaboAnalyst to identify cellular metabolites associated with drug chemosensitivity.

**Results:**

A total of 1,624 metabolic features were detected across the leukemic cell lines. Of these, 187 were annotated to known metabolites. With respect to doxorubicin, we observed significantly greater abundance of a carboxylic acid (1-aminocyclopropane-1-carboxylate) and several amino acids in resistant cell lines. Pathway analysis found enrichment of several amino acid biosynthesis and metabolic pathways. For cytarabine resistance, nine annotated metabolites were significantly different in resistance *vs*. sensitive cell lines, including D-raffinose, guanosine, inosine, guanine, aldopentose, two xenobiotics (allopurinol and 4-hydroxy-L-phenylglycine) and glucosamine/mannosamine. Pathway analysis associated these metabolites with the purine metabolic pathway.

**Conclusion:**

Overall, our results demonstrate that metabolomics differences contribute toward drug resistance. In addition, it could potentially identify predictive biomarkers for chemosensitivity to various anti-leukemic drugs. Our results provide opportunity to further explore these metabolites in patient samples for association with clinical response.

## Introduction

Acute myeloid leukemia (AML) is a hematological disease resulting from proliferation and expansion of malignant myeloid cells. Successful treatment of AML requires the achievement of complete remission. Therefore, chemotherapeutic regimens for treatment of AML are very aggressive. The most commonly used induction treatment regimen for AML is the 7 + 3 model, named for the schedule of a 7-day infusion cycle of cytarabine combined with a 3-day intravenous push of an anthracycline agent, most often doxorubicin, daunorubicin, or idarubicin (1). The 7 + 3 regimen is widely used today and has changed very little for several decades. Despite being so well established, the rates of achieving complete remission with the 7 + 3 regimen are approximately 70% in patients <60 years old and 50% in patients >60 years old (2–4). Five-year survival rates are also suboptimal at approximately 60% for pediatric patients and 24% for adult patients (5). One of the major causes of these poor treatment outcomes is the development of resistance to the chemotherapeutic agents used in standard treatment regimens. Cancer cells that are able to adapt and resist induction therapy are frequently the cause of relapse, and often lead to a worse prognosis for patients. Because of the prevalence of chemotherapeutic resistance and subsequently poorer treatment outcomes, there is an ongoing effort to understand the molecular mechanisms that may be contributing to variations in response to therapy.

Changes to cellular metabolism have been associated with cancer development and tumor growth for multiple cancer types. The growing field of metabolomics and its approaches allows full metabolic profile evaluation in AML patients and leukemic cell cultures to explore the relationship between metabolism and resistance. Metabolomics studies of leukemia cell lines have been targeted to an identify metabolic pathways of resistance in multiple forms of leukemia (6–8). One study specifically investigating chemo-sensitivity has further explored the alterations to glutamine, glucose, fatty acid metabolism, and oxidative phosphorylation in cells resistant to commonly used chemotherapy (9). However, global metabolomic evaluation in this context is virtually unexplored. In this study, we established the global metabolome for seven commercially available AML cell lines with varying drug chemo-sensitivity in order to identify metabolic pathways with potential role in development of drug resistance. We anticipate that by performing metabolomic profiling of AML cell lines and comparing these profiles to previously generated dose response data, then we will find significant association of metabolite abundance with dose response.

## Methods

### Cell Lines and Cellular Cytotoxicity Assays

Seven AML cell lines were used for metabolomics profiling. AML cell lines HL-60 (ATCC CCL-240), MV-4-11 (ATCC CRL-9591), AML-193 (ATCC CRL-9589), Kasumi-1 (ATCC CRL-2724), KG-1 (ATCC CCL-246), and THP-1 (ATCC TIB-202) were purchased from the American Type Culture Collection (ATCC, Manassas, VA). ME-1 (ACC-537) cell line was purchased from DSMZ (Braunschweig, Germany). All cell lines were cultured using appropriate growth media recommended by the cell line supplier. Cell cultures were incubated at 37°C with a CO_2_ concentration of 5%. MTT assay was used to measure cytotoxicity as described previously (10). Briefly, AML cell lines were maintained in the media as per the recommendations. Cells were treated with varying concentrations of cytarabine (ranging 200–0.01 µM) and doxorubicin (10–0.1 µM). Cell viability was measured using the MTT reagent 48h post-treatment. Absorbance was measured at 570nm using Synergy plate reader (BioTek, USA). Area under the curve (AUC) of the cell viability curve was calculated using the trapezoidal method using GraphPad Prism software.

### Sample Preparation for Metabolomics Study

Cells (10^6^) were 3x washed with 40 mM ammonium formate solution followed by addition of 50 μL of a 5 mM ammonium acetate. Bead Beater (Fastprep 96, MPBio, Santa Ana, CA) was used to homogenize the cells at 1,800 rpm for 30 s, followed by incubation at 4°C for 15 min. Homogenization was repeated two more times with incubation at 4°C for 15 min. Two microliters of internal standard solution (Creatine-D3, L-Leucine-D10, L-Tryptophan-D3, Caffeine-D3, L-Tryptophan-2,3,3-D3, Succinic Acid-D4, and Salicylic Acid-D4) and 1 mL of 80% methanol in water solution were added to each sample. Samples were mixed using the Bead Beater at the previous settings and incubated at 4°C for 10 min. Following centrifuging at 200,000xg for 10 min at 4°C, 1 mL supernatant were collected and dried using nitrogen gas (N2 Dryer, Organomation Associates Inc.). Samples were reconstituted using 100 μL reconstitution solution (Phenylalanine_t-BOC, Tryptophan_t-BOC, Tyrosine_t-BOC in 0.1% formic acid in water) on ice for 15 min, following centrifugation, supernatants loaded for ultra-high performance liquid chromatography–mass spectrometry (UHPLC-MS).

### Global Metabolomics

Untargeted metabolomics profiling was performed with a Thermo Ultimate 3000 UHPLC and Thermo Q-Exactive Orbitrap mass spectrometer (Thermo, San Jose, CA) with the assistance of the Southeast Center for Integrated Metabolomics (SECIM) Core Lab 1 at the University of Florida. All samples were run in positive and negative ionization through heated electrospray as separate injections. Mass spectra analysis had a mass resolution of 35,000 at m/z 200. Separation was achieved on an ACE 18-pfp 100 × 2.1 mm, 2 μm column. Mobile phase A was 0.1% formic acid in water, and mobile phase B was acetonitrile. The flow rate was 350 μL/min, and column temperature was set to 25°C. Two microliters was injected for positive ionization and 4 μL for negative ionization.

### Statistical Analysis

Mass spectra output files were converted to mzxml file format using MS Convert software (ProteoWizard 3.0). MZmine (freeware) was used to identify features, deisotope, align features and perform gap filling to fill in any features that may have been missed in the first alignment algorithm. All adducts and complexes were identified and removed from the data set. Positive and negative ionization datasets were merged to be used for statistical analyses. Peak intensity values for each cell line were grouped according to assigned cytarabine and doxorubicin sensitivity groups (sensitive or resistant) in separate datasets for categorical analysis. Missing value estimation was performed by removing metabolites missing over 30% of values among the samples and replacing the remaining missing values with a small value (half of the minimum positive value in the original data). Data processing included normalization by sum, log transformation, and Pareto scaling.

Processed and normalized metabolomics data were then analyzed using categorical and continuous forms of the chemosensitivity variables, chemosensitivity group assignment, and cell viability AUC, respectively. Categorical statistical analysis was performed on the MetaboAnalyst 4.0 web-based analysis platform (11) using univariate, multivariate, and clustering methods. Univariate analyses included t-test and fold change analysis. Significance threshold was set at p-value <0.05. Multivariate analyses included principal component analysis (PCA) and partial least square discriminatory analysis (PLSDA). Clustering analysis included generation of unsupervised heatmaps. Continuous statistical analysis was performed on GraphPad Prism software using Pearson correlation analysis. Significance threshold was set at p-value <0.05.

### Pathway Analysis

Peak intensity values for metabolites with significantly different abundance AML cell lines were imported into MetaboAnalyst pathway analysis function, which performed pathway enrichment and topology analysis. Metabolite identifiers were converted as necessary according to synonyms listed in the human metabolome database (HMDB). The pathway impact measurement represented the sum of importance measures, generated by topology analysis, of significant metabolites normalized by importance measures of all metabolites in the associated pathway. The datasets generated and/or analyzed during the current study are available from the corresponding author on request. Additionally, we also cross-referenced the significant metabolites identified in the study with drug candidate analysis using Human Metabolome Database (HMDB) (hmdb.ca) database.

## Results

### AML Cell Line Chemo-Sensitivity to Cytarabine and Doxorubicin

**Figure 1:**
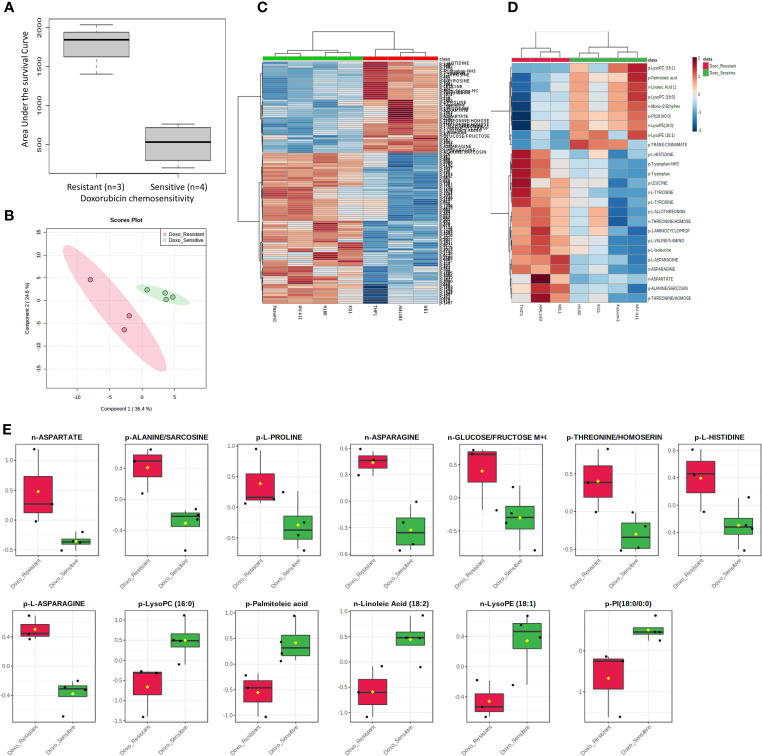
Metabolome analysis by doxorubicin *in vitro* chemosensitivity in AML cell lines. **(A)** Box plot showing differential AUC values between doxorubicin sensitive and resistant cell lines. **(B)** Multivariate metabolomics analysis of AML cell line doxorubicin chemosensitivity groups. PLSDA plot of cell samples shows global separation by doxorubicin chemosensitivity (Sensitive n=4 and Resistant n=3). **(C)** Clustering metabolomics analysis of AML cell line doxorubicin chemosensitivity groups. Heatmap shows relative abundance patterns of 122 cell metabolites (annotated and un-annotated) with significantly different abundance between groups. Clustering within the heatmap shows a clear distinction of several metabolites between the sensitive and resistant groups. **(D)** Abundance patterns of top 25 annotated metabolites are shown in this Heatmap. **(E)** Box plots of selected metabolites showing abundance by drug sensitivity groups. p, positive and n, negative ionization set.

Cell lines were categorized as sensitive or resistant to treatment based on area under the survival cure (AUC) values for cytarabine and doxorubicin obtained from *in vitro* cytotoxicity determined using MTT assays. For doxorubicin the HL-60, MV-4-11, Kasumi-1, and KG-1 (AUC below 1000) were classified as sensitive and AML-193, ME-1, and THP-1 were classified as resistant (sensitive *vs*. resistant cell lines, **Figure 1A**). With respect to cytarabine AML-193, Kasumi-1, and THP-1 cell lines with a cytarabine cytotoxicity AUC value greater than 12,000 were considered resistant to cytarabine, and cell lines HL-60, MV-4-11, KG-1, and ME-1 with a cytotoxicity AUC value less than 12,000 were categorized as sensitive to cytarabine (sensitive *vs*. resistant cell lines, **Figure 3A**). Characterization and cytotoxicity results for these cell lines are listed in **Table 1**.

**Table 1:** Characterization of acute myeloid leukemia (AML) cell lines based on cytarabine and doxorubicin chemosensitivity.

Cell Line	Cytarabine cytoxicity AUC	Cytarabine Sensitivity Group	Doxorubicin cytoxicity AUC	Doxorubicin Sensitivity Group	Cytogenetic Profile
ME-1	6497	Sensitive	2035	Resistant	inv(16)(p13q22), CBFB-MYH11 gene fusion
AML-193	12988	Resistant	1843	Resistant	+der(17)t(17;17)(p13.1;q21.3)
THP-1	17170	Resistant	1403	Resistant	t(9;11)(p21;q23), RUNX1/AML1-RUNX1T1/ETO gene fusion; TP53 mutation
HL-60	4597	Sensitive	673.4	Sensitive	CDKN2A, NRAS, TP53 mutations
MV-4-11	5011	Sensitive	201.9	Sensitive	FLT3-ITD mutation; t(4;11)(q21;q23), MLL-AF4 gene fusion
KG-1	5939	Sensitive	762.5	Sensitive	NRAS, P53 mutation; RB1 rearrangement
Kasumi-1	14713	Resistant	390.1	Sensitive	t(8;21)(q22;q22), RUNX1/AML1-RUNX1T1/ETO gene fusion; TP53 mutation

### Metabolomics Profiling

Global metabolomics profiling of the AML cell lines was conducted using UHPLC-MS with the assistance of the SECIM Core Lab at the University of Florida. Global metabolomes were generated with the application of both positive and negative ionization settings separately. A total of 1,624 metabolic features were detected across the leukemic cell lines of which 187 were annotated to known metabolites as per the SECIM metabolite library.

### Metabolites and Pathways Associated With Doxorubicin Chemosensitivity in AML Cell Lines

We utilized MetaboAnalyst software (11) to perform categorical analysis of all 1,624 features to identify metabolome features with significantly different abundance between AML cell lines based on doxorubicin sensitivity. This analysis identified 122 metabolites with significantly differential abundance between the groups (p<0.05) and included 23 annotated metabolites, all of which demonstrated greater abundance in doxorubicin resistant cell lines (**Table 2**). Heatmap of the 122 metabolites (annotated and unannotated) found to be significantly different by doxorubicin sensitivity groups is shown in **Figure 1C**, which shows clear clustering of metabolites by doxorubicin chemosensitivity. **Supplementary Figure 1** shows the heatmap of global metabolome of AML cell lines by drug sensitivity. Multivariate analyses using PLSDA analysis though showed separation between the cell line global metabolomes (**Figure 1B**). We also performed another categorical analysis focusing on metabolomics data consisting of 187 annotated features, and identified 11 annotated features significantly differed by doxorubicin resistance in this analysis (t-test p <0.05, fold change threshold of 2). Of these, five overlapped (L-asparagine, alanine/sarcosine, asparagine, aspartate and threonine/homoserine) with the global analysis and six additional metabolites (Lipids and fatty acids), all with lower abundance with doxorubicin resistance, identified included: palmitoleic acid, LysoPE(18:1), linoleic acid, LysoPC(16:1), PI(18:0/0:0), LysoPC (16:0). **Figure 1D** shows the results of the multivariate analysis using PLSDA for the annotated metabolites. In addition, we also performed a correlation analysis (Pearson’s correlation) between AUC levels and annotated metabolite levels and identified 25 annotated metabolites to be significantly correlated with AUC values (p<0.05). Among these metabolites, 24 were positively correlated and 1 metabolite [lysophosphatidylcholine 16:1 (LysoPC 16:1)] was negatively correlated with AUC values. Positively correlated metabolites included a carboxylic acid (1-aminocyclopropane-1-carboxylate) and several amino acids (listed in **Table 2**). Box plots depicting differential abundance of selected metabolites by the doxorubicin resistance from analysis described above are shown in **Figure 1E**.

**Table 2:** List of metabolites significantly associated with doxorubicin resistance in 7 AML cell lines.

Metabolite	Ionization Set	Classification	Associated Pathway	p-value (t-test)	Fold Change	Correlation (Pearson r)	Correlation p-value
L-Valine/5-Aminopentanoate/L-Norvaline	Positive	Amino Acids	Pantothenate and CoA biosynthesis; valine, leucine, and isoleucine biosynthesis and degradation; Lysine degradation	**0.0009**	2.33	0.957	**0.0007**
Leucine	Positive	Amino Acids	Aminoacyl-tRNA biosynthesis; valine, leucine, and isoleucine biosynthesis and degradation	**0.005**	2.34	0.827	**0.022**
Threonine/Homoserine	Negative	Amino Acids	Aminoacyl-tRNA biosynthesis; Cysteine and methionine metabolism	**0.0053**	2.40	0.912	**0.004**
1-Aminocyclopropane-1-Carboxylate	Positive	Amino Acids	N/A	**0.0042**	2.43	0.931	**0.002**
Phenylalanine	Positive	Amino Acids	Phenylalanine, tyrosine, and tryptophan biosynthesis; Nitrogen metabolism; Phenylalanine metabolism; Aminoacyl-tRNA biosynthesis	**0.0169**	2.49	0.764	**0.046**
Trigonelline	Positive	Alkaloids	Nicotinate and nicotinamide metabolism	**0.0326**	2.54	0.735	0.060
Phenylalanine-HCOOH	Positive	Amino Acids	N/A	**0.0161**	2.55	0.758	**0.048**
L-Isoleucine	Positive	Amino Acids	Aminoacyl-tRNA biosynthesis; valine, leucine, and isoleucine biosynthesis and degradation	**0.0016**	2.61	0.929	**0.003**
L-Tyrosine	Negative	Amino Acids	Phenylalanine, tyrosine, and tryptophan biosynthesis; Tyrosine metabolism	**0.0006**	2.61	0.868	**0.011**
L-Tyrosine	Positive	Amino Acids	Phenylalanine, tyrosine, and tryptophan biosynthesis; Tyrosine metabolism	**0.0021**	2.64	0.872	**0.011**
Tryptophan	Positive	Amino Acids	Tryptophan Metabolism, Nitrogen metabolism, Aminoacyl-tRNA metabolism	**0.003**	2.70	0.824	**0.023**
L-Allothreonine	Positive	Amino Acids	Glycine, serine, and threonine metabolism	**0.0095**	2.79	0.903	**0.005**
Tryptophan-NH3	Positive	Amino Acids	Tryptophan Metabolism, Nitrogen metabolism, Aminoacyl-tRNA metabolism	**0.003**	2.81	0.816	**0.025**
Alanine/Sarcosine	Positive	Amino Acids	Taurine and hypotaurine metabolism; Alanine, aspartate, and glutamate metabolism; Glycine, serine, and threonine metabolism	**0.0028**	3.04	0.976	**0.000**
L-Asparagine	Negative	Amino Acids	Alanine, aspartate and glutamate metabolism; Nitrogen Metabolism; Cyanoamino acid Metabolism	**0.0003**	3.14	0.962	**0.001**
Threonine/Homoserine	Positive	Amino Acids	Aminoacyl-tRNA biosynthesis; Cysteine and methionine metabolism	**0.0072**	3.29	0.952	**0.001**
L-Histidine	Positive	Amino Acids	beta-Alanine metabolism, Nitrogen metabolism, Histidine metabolism	**0.0147**	3.38	0.730	0.062
L-Proline	Positive	Amino Acids	Arginine and proline metabolism	**0.0286**	3.44	0.789	**0.035**
Glucose/Fructose	Negative	Sugars	Starch and sucrose metabolism; Galactose Metabolism; Pentose Phosphate Pathway; Amino sugar and nucleotide sugar metabolism	**0.0378**	3.55	0.626	0.133
L-Asparagine	Positive	Amino Acids	Alanine, aspartate and glutamate metabolism; Nitrogen Metabolism; Cyanoamino acid Metabolism	**0.0004**	3.96	0.972	**0.000**
L-Histidine	Negative	Amino Acids	beta-Alanine metabolism, Nitrogen metabolism, Histidine metabolism	**0.0403**	4.53	0.774	**0.041**
Aspartate	Positive	Amino Acids	Alanine, aspartate and glutamate metabolism; Nitrogen Metabolism; Cyanoamino acid Metabolism	**0.0392**	5.37	0.786	**0.036**
Aspartate	Negative	Amino Acids	Alanine, aspartate and glutamate metabolism; Nitrogen Metabolism; Cyanoamino acid Metabolism	**0.019**	5.68	0.819	**0.024**
Glycine	Positive	Amino Acids	Cyanoamino acid metabolism, Glutathione metabolism, Nitrogen metabolism, Primary bile acid biosynthesis, Glycine, serine, and threonine metabolism	0.055	6.902	0.828	**0.021**
LysoPC(16:1)	Positive	Lipids	Lysophospholipid Metabolism	0.088	0.202	-0.809	**0.028**
L-Glutamine	Negative	Amino Acids	D-Glutamine and D-glutamate metabolism; Alanine, aspartate, and glutamate metabolism	0.062	2.524	0.800	**0.031**
L-Serine	Positive	Amino Acids	Cyanoamino acid metabolism; Sulfur metabolism; Glycine, serine, and threonine metabolism; Cysteine and methionine metabolism; Aminoacyl-tRNA biosynthesis	0.085	7.592	0.795	**0.033**
Glycerol	Positive	Sugar Alcohols	Glycerophospholipid metabolism	0.181	1.565	0.764	**0.046**

P vlaues <0.05 are in bold; p values >0.05 are not in Bold.

**Figure 2:**
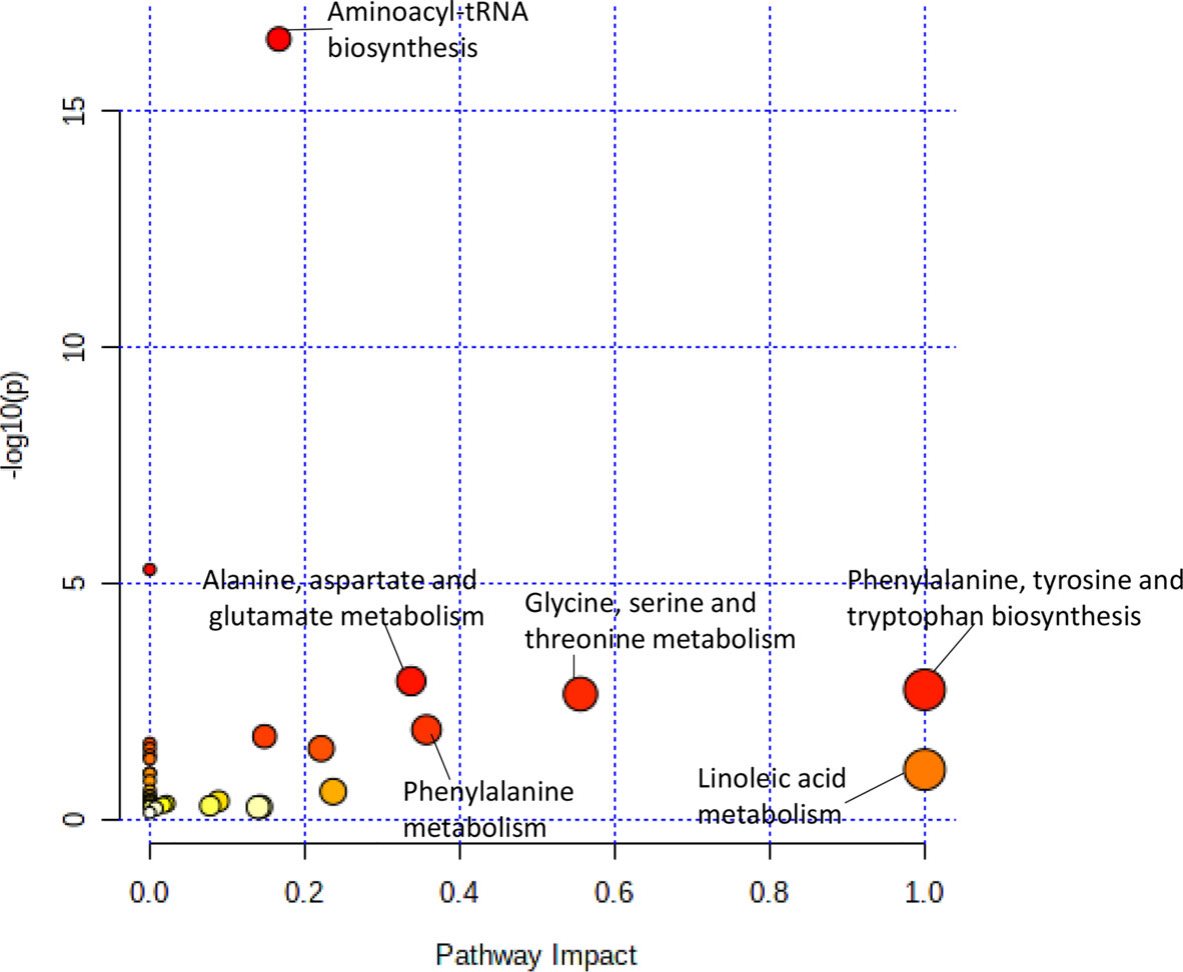
Pathway analysis of metabolites significantly associated with doxorubicin AUC. Metabolic pathways associated with doxorubicin sensitivity showed a greater variety of amino acid synthesis and metabolism pathways.

The pathway analysis used a pathway library in the MetaboAnalyst platform, which contained 80 known human metabolic pathways from the Kyoto Encyclopedia of Genes and Genomes (KEGG) database (12–14). A false discovery rate (FDR) significance threshold was set at less than 0.05. The pathway analysis of metabolites associated with doxorubicin chemosensitivity identified five significant metabolic pathways (**Figure 2**). These pathways included a variety of aminocyl-tRNA biosynthesis and metabolic pathways, for valine, leucine, and isoleucine biosynthesis; alanine, aspartate, and glutamate metabolism; phenylalanine, tyrosine, and tryptophan biosynthesis; and glycine, serine, and threonine metabolism (**Supplementary Table 1**). Intriguingly, aspirin decreases the blood levels of five of the metabolites that we identified as overexpressed in doxorubicin resistant AML cell lines: glycine, L-asparaginase, L-histidine, L-serine, and L-glutamine (15) obtained from hmdb.ca).

### Metabolites and Pathways Associated With Cytarabine Chemosensitivity in AML Cell Lines

**Figure 3:**
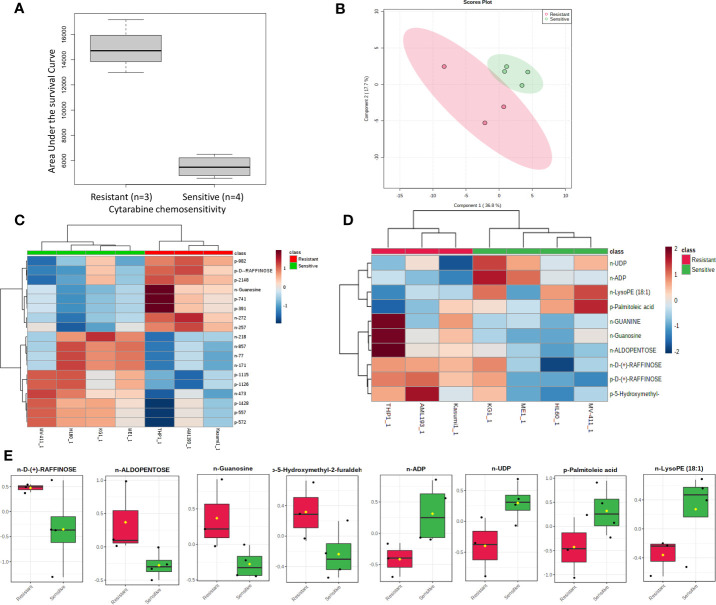
Metabolome analysis by cytarabine *in vitro* chemosensitivity in AML cell lines. **(A)** Box plot showing differential AUC values between cytarabine sensitive and resistant cell lines. **(B)** Multivariate metabolomics analysis of AML cell line cytarabine chemosensitivity groups. **(B)** PLSDA plot of cell samples shows global separation by cytarabine chemosensitivity (Sensitive n=4 and Resistant n=3). **(C)** Clustering metabolomics analysis of AML cell line cytarabine chemosensitivity groups. Heatmap shows relative abundance patterns of 18 cell metabolites (annotated and un-annotated) with significantly different abundance between groups. Clustering within the heatmap shows a clear distinction of several metabolites between the sensitive and resistant groups. **(D)** Clustering metabolomics analysis of only annotated metabolites heatmap shows relative abundance patterns of top 10annotated cell metabolites with differential abundance between groups. **(E)** Box plots of selected metabolites showing abundance by drug sensitivity groups. p= positive and n=negative ionization set.

**Figure 4:**
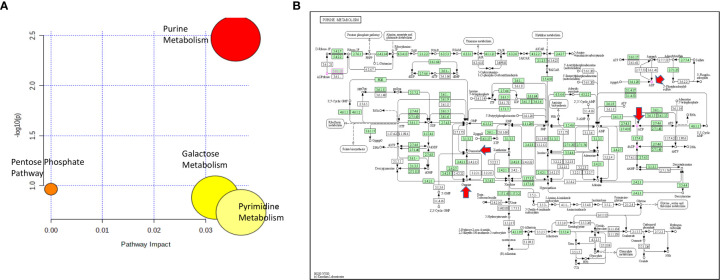
**(A)** Pathway analysis of metabolites significantly associated with cytarabine AUC. **(B)** The lone significantly associated metabolic pathway was purine metabolism. Guanine, ADP and guanosine are highlighted by red arrows in the KEGG purine metabolism pathway.

For cytarabine chemosensitivity, a correlation analysis was conducted to test the relationship of the abundance of known metabolites in the AML cell lines with the cytarabine cytotoxicity AUC data. A total of nine known metabolites were found to be significant, with all nine metabolites positively associated with cytotoxicity AUC (p<0.05). These included three nucleosides (inosine, positive, and negative ion forms of guanine), the carbohydrate aldopentose, two xenobiotics (allopurinol and 4-hydroxy-L-phenylglycine), and the amino sugar glucosamine/mannosamine (**Table 3**). Categorical analysis of the global metabolome data using Metaboanalyst found a total of 18 metabolites (annotates and unannotated) to be significantly different by cytarabine chemosensitivity (p-value < 0.05) and included only two annotated metabolites, the nucleoside guanosine and the sugar D-Raffinose. **Figure 3C** shows a heatmap of the 18 significantly different metabolites (2 annotated and 16 unannotated) by cytarabine chemosensitivity group. The PLSDA multivariate modeling showed separation of two groups (**Figure 3B**). Evaluation of only annotated metabolites was also performed separately; the corresponding heatmap showing differences in abundance of top 10 annotated metabolites is shown in **Figure 3D** (these included UDP, ADP, Lyso-PE, palmitoleic acid, guanine, aldopentose and 5-hydroxymethyl furaldehyde in addition to guanosine and D-raffinose). The global metabolome by AML cell lines is shown in **Supplementary Figure 1**. Box plots for D-raffinose, aldopentose, guanosine, 5’ hydroxymehtyl-2 furaldehyde, which demonstrated higher levels in cytarabine resistant cell lines and ADP, UDP, palmitoleic, and Lyso-PE with higher abundance in the cytarabine resistant cell lines are shown in **Figure 3E**. The pathway analysis of the metabolites significantly associated with cytarabine cell viability AUC identified purine metabolism (**Figure 4A**) as the only significantly associated pathway. Pentose phosphate metabolism, pyrimidine metabolism, and galactose metabolism pathways were also enriched but were not statistically significant. **Figure 4B** shows KEGG Purine metabolic pathway with guanosine, guanine, and ADP highlighted. Drugs known to impact these metabolites include: cocaine which increases guanine and guanosine in blood (16), aspirin increases guanosine and inosine in blood (17), and opioid dependence associates with increase guanine and decreased guanosine in blood (18).

**Table 3:** List of cellular metabolites significantly associated with cytarabine AUC values by Pearson correlation and categorical analysis in AML cell lines.

Metabolite	Ionization Set	Classification	Associated Pathway	Pearson r	p-value
Guanosine	Negative	Nucleosides	Purine metabolism	0.885	0.008
Aldopentose	Negative	Carbohydrates	Pentose Phosphate Pathway	0.866	0.012
4-Hydroxy-L- Phenylglycine	Positive	Xenobiotics	N/A	0.851	0.015
Guanine	Negative	Nucleosides	Purine metabolism	0.831	0.021
Guanine	Positive	Nucleosides	Purine metabolism	0.830	0.021
D-Raffinose	Negative	Sugars	Galactose metabolism	0.808	0.028
Glucosamine /Mannosamine	Positive	Sugars	Amino sugar and nucleotide sugar metabolism	0.804	0.029
Inosine	Negative	Nucleosides	Purine metabolism	0.797	0.032
Allopurinol	Positive	Xenobiotics	N/A	0.795	0.033
**Metabolite**	**Ionization Set**	**Classification**	**Associated Pathway**	**Fold Change**	**p-value (t-test)**
D-Raffinose	Negative	Sugars	Galactose metabolism	3.49	0.03
Guanosine	Negative	Nucleosides	Purine metabolism	2.72	0.04

## Discussion

In this study, we evaluated the global metabolic profiles of seven AML cell lines with varying sensitivity to two of the most commonly used chemotherapeutic agents used for treatment of AML: cytarabine and doxorubicin. Though the chemosensitivity grouping according to cytarabine or doxorubicin was not able to define the global metabolome for the AML cell lines in unsupervised multivariate analysis, we identified metabolites and metabolic pathways that showed significant differential abundance based on differences in chemosensitivity to both cytarabine and doxorubicin.

With respect to cytarabine chemosensitivity, we identified a greater abundance of multiple nucleosides in AML cell lines that are more resistant to cytarabine relative to those that are sensitive. Pathway analysis further related these nucleosides to a significantly impacted purine metabolism pathway in the cytarabine resistant cell lines. An increase in nucleosides may be related to the pathway of cytarabine activation, and specifically indicate an established mechanism for cytarabine resistance (19, 20). Cytarabine is administered in the form of cytosine arabinoside, and functions as an analog to deoxycytidine. Once cytarabine is transported into the cell, it is rapidly phosphorylated multiple times to form cytarabine triphosphate, its active metabolite. However, multiple enzymes can reverse this process and inactivate cytarabine at different steps. The 5’-nucleotidase family of enzymes is responsible for dephosphorylation of nucleotide bases to nucleosides, which includes cytarabine monophosphate as a substrate. The chemotherapeutic effect of cytarabine requires phosphorylation to its triphosphate form in order to be incorporated into the DNA to carry out its function (21, 22). By reversing the initial step of phosphorylation, the increased activity of 5’-nucleotidases can cause increased resistance to cytarabine through inactivation and could explain the increased abundance of nucleosides in resistant AML cell lines.

With respect to doxorubicin, several amino acids showed a significantly higher intracellular abundance in doxorubicin resistant cell lines. The importance of amino acids for cancer cell proliferation and survival is well established (23, 24) with amino-acid metabolic pathways shown to be rewired in cancer cells to keep up with the demands for energy production and protein biosynthesis. In this study, an increased abundance of valine, isoleucine, tyrosine, alanine/sarcosine, proline, and phenylalanine was associated with doxorubicin resistance. A lower abundance of several of these amino acids has been observed in serum from AML patients as compared to healthy controls, suggesting amino acid depletion in extracellular environment potentially due to increased cellular uptake for various metabolic processes (25, 26). Intriguingly, as mentioned above, asprin decreases the blood levels of several metabolites when cross checked with the human metabolome database. Our results are concordant with multiple reports showing that aspirin can suppress chemoresistance in various malignancies (27–32). A clinical trial is currently evaluating the efficacy of aspirin and tamoxifen in combination with doxorubicin as part of standard AC-T chemotherapy for treatment of high risk ER+ or ER- breast cancer (NCT04038489 clinicaltrials.gov).

The increased abundance of asparagine in the doxorubicin resistant cells seen in our results shows a classic example of altered amino acid metabolism seen in acute leukemia. Acute lymphoblastic leukemia (ALL) cells have an increased dependence on exogenous asparagine as an essential amino acid due to decreased activity of asparagine synthetase. This results in a powerful therapeutic opportunity targeted by asparaginase, which depletes the circulating asparagine and starves the leukemic cells of the asparagine they require for survival (23). While the depletion of circulating asparagine is not as common in AML therapy, there is growing evidence to show that AML cells have a similar dependence on asparagine and vulnerability to asparaginase (33, 34). Treatment of AML cell lines and primary cells with asparaginase has shown increased sensitivity to the drug in cells with monosomy 7 compared to those without (35). Our results suggest that this pattern of asparagine importance may be further increased in chemoresistant cell lines. However, further investigation of ASNS activity and asparagine efficacy in these cell lines is needed to confirm this. A higher intracellular abundance of glutamine was observed in doxorubicin resistant cell lines; glutamine serves as a key component for energy production and redox regulation necessary for sustaining cell proliferation and survival in cancer cells and AML cell in particular (33, 36, 37). However, a recent metabolomics study focused on the rewiring of metabolism that occurs in chemoresistant leukemic cells showed that resistant cells display a significantly reduced dependence on glutamine for survival (9). This suggests that glutamine may not be used primarily for its contributions to energy production in chemoresistant cells. Glutamine has an additional role in leukemic cells as a component of glutathione synthesis, an important factor for protecting cells against reactive oxygen species (ROS). In fact, in a recent report by Emadi et al. (2020) in complex karyotype AML, it was observed that asparaginase mediated glutamine depletion and subsequent inhibition of 4EBP1 and reduced MCL1 expression are the mostly likely underlying mechanisms for synergistic effect observed in BCL2 inhibition with venetoclax and asparaginase combination (38). FLT3 inhibitor quizartinib has been shown to be a significant inhibitor of glutamine based generation of glutathione by inhibiting cellular glutamine uptake (39). The increase in glutamine abundance in resistant AML cells, despite their reduced dependence on it for survival, may be explained by its role in glutathione production for ROS protection. Further study of glutamine metabolism is needed to fully understand its role in chemoresistant leukemia cells. Beyond the contributions of glutamine, there are additional amino acids involved in the metabolic pathway for glutathione biosynthesis. Our results show that glycine, one of these amino acids, also has increased abundance in doxorubicin resistant AML cell lines. Glycine is involved in the final step of glutathione synthesis. The enzyme glutathione synthase adds glycine to the C-terminal of L-gamma-glutamyl-L-cysteine to form glutathione (40). Similar to glutamine, the increased abundance of glycine in chemoresistant AML cell lines may be explained by its contribution to increased glutathione synthesis for additional protection from oxidative stress. To supplement this further, the non-essential amino acid serine is a generator for glycine through the one carbon donor pathway (24). Our analysis shows an increase in cellular serine abundance as well in doxorubicin resistant AML cell lines. A substantial amount of serine is used for glycine synthesis through this pathway, and its increased abundance in chemoresistant cell lines may be contributing to the increase in glycine synthesis and abundance as well.

Of particular interest were also metabolites with a role in altered lipid and fatty acid metabolism associated with respect to doxorubicin chemosensitivity (low levels of LysoPC 16:1 and high levels of glycerol in doxorubicin resistant AML cell lines). Alterations to lipid metabolism are a common feature across multiple cancer types (41–46), and a growing number of studies have shown its importance for AML as well (7, 47–49). One of the key alterations to lipid metabolism in AML is an increased dependence on lipid catabolism through fatty acid oxidation (FAO). The reduction in LysoPC abundance and the increase in glycerol may indicate this increase in lipid and triglyceride catabolism. FAO is a mechanism for providing acetyl-CoA to the citric acid cycle. This leads to increased citrate generation, which can be used as a key component for fatty acid synthesis. The repetitive breakdown and subsequent re-synthesis of fatty acids is known as the “futile metabolic cycle” that is a unique feature of cancer cell metabolism, which can provide cancer cells with protection against oxidative stress and initiation of apoptosis (50). Further evidence supporting the importance of fatty acid oxidation in AML is seen in studies relating to the enzyme carnitine palmitoyltransferase I (CPT1), which catalyzes the rate limiting step of the carnitine shuttle component of fatty acid oxidation (9, 51, 52). Overall, the reduced LysoPC 16:1 and increased glycerol seen in the doxorubicin resistant AML cell lines may be indicative of an increased rate of lipid and triglyceride catabolism contributing to fatty acid oxidation.

Overall, our study demonstrates that significantly different patterns of metabolite abundance can be found when comparing AML cell lines based on sensitivity to chemotherapeutic agents commonly used for AML treatment. These metabolites can be linked to several metabolic pathways known to be related to AML disease progression and chemotherapy resistance. AML cell lines that are resistant to cytarabine therapy show a significant alteration to purine metabolism, with a higher abundance of nucleosides related to this pathway. Resistance to doxorubicin in AML cell lines was found to be related to more widespread changes in metabolic pathways, including lipid catabolism, increased fatty acid oxidation, increased amino acid uptake, increased glycine and serine metabolism, and increased glutathione synthesis.

While the results of cell line metabolomics analysis are promising, they require a follow-up study on a larger scale. Ideally, a large patient cohort study integrating metabolomics data in cell samples with additional patient omics data, such as genomics, transcriptomics, and proteomics, could clarify the full picture of what alterations can contribute to the chemoresistant phenotype in leukemic cells. However, this study helps support our understanding of what metabolic processes are linked to resistance to commonly used chemotherapeutic agents. Expanding our knowledge of this relationship can potentially improve our systems of selecting initial therapies and may lead to new targets for improving AML treatment.

## Data Availability Statement

The data supporting the conclusion of this article are available upon request from the authors.

## Author Contributions

BS and JKL contributed to study design. BS, NB, MS, JG-C did experiments and generated data. BS, JG-C, SP, TG and JKL performed data analysis and interpretation. BS and JKL drafted the manuscript. All authors provided critical review of the manuscript and have approved it for publication. All authors contributed to the article and approved the submitted version.

## Funding

This research was supported by NIH under the award numbers: NCI-R01CA132946.

## Conflict of Interest

The authors declare that the research was conducted in the absence of any commercial or financial relationships that could be construed as a potential conflict of interest.
